# Characteristics and trends of traumatic injuries in children visiting emergency departments in South Korea: A retrospective serial cross-sectional study using both nationwide-sample and single-institutional data

**DOI:** 10.1371/journal.pone.0220798

**Published:** 2019-08-09

**Authors:** Michael Seungcheol Kang, Han-Soo Kim

**Affiliations:** 1 Department of Orthopaedic Surgery, Asan Medical Center Children’s Hospital, Seoul, Republic of Korea; 2 Department of Orthopaedic Surgery, Seoul National University Hospital, Seoul, Republic of Korea; University of Texas Health Science Center at Houston, UNITED STATES

## Abstract

We investigated the incidences and characteristics of pediatric traumatic injuries requiring emergency department visits, through a complementary approach using both nationwide-sample and single-institutional data. Data for children (aged <15 years) identified with traumatic injuries during a 10-year period from the Korean National Health Insurance Sharing Service (n = 35,064 among 10,114,909 randomly sampled cases from the claim records of the National Health Insurance) and the authors' institute (n = 39,228) were retrospectively reviewed. The incidences and characteristics of the injuries were investigated using both datasets; additionally, detailed information regarding the injury environments was investigated using the single-institutional data. The findings were similar across both datasets. The incidence of injuries increased during the study period; the head was most commonly injured, whereas the trunk or proximal extremities were rarely injured; low-energy head injuries accounted for >50% of the cases in children aged <5 years, although the incidences of lower-extremity injuries and fractures increased in older children. Single-institutional data demonstrated that the proportion of indoor playground and trampoline-related injuries increased rapidly during the study period, and outdoor injuries and seasonal variation (with peak incidences in May and June) were more prominent in older children. Based on similarities between both datasets, the detailed results regarding pediatric traumatic injuries obtained from the single-institutional data could be generalized nationally with adequate external validity. To prevent traumatic injuries, it may be more effective to wear protective equipment covering the head and distal extremities rather than the trunk or proximal extremities; simple clothing, such as caps, could prevent many injuries in preschoolers. Among older children, safety guidelines for outdoor sports/leisure activities are needed. The increase in pediatric traumatic injuries may be partially explained by the increased availability of indoor playgrounds and installation of trampolines. Stricter adherence to the preventive guidelines is needed.

## Introduction

Traumatic injuries are a major cause of impairment or death in children [1]. These injuries occur in various places during various activities [2] and change in their patterns over time [3–6]. For example, sports participation is promoted to increase physical activity among children in most developed countries [7, 8], and participation in sports and leisure activities is increasing among this population [4, 5, 9]. By contrast, other activities in daily life, such as walking, tend to decrease [5, 6, 10]. Therefore, a comprehensive survey of pediatric traumatic injuries that is constantly updated is needed.

A substantial proportion of pediatric traumatic injuries is preventable [2]; therefore, many countries are using strategies, including educational programs, to prevent these [11]. Although prevention of injuries in each country based on investigations performed in each country is likely to be more effective, few countries with a specific nationwide surveillance system have reported comprehensive studies [12]. Instead, the results of a comprehensive nationwide study performed in some specific countries are likely to be applicable to many other countries with similar socioeconomic and environmental conditions, as general lifestyles and medical environments become increasingly similar globally [1]. Nevertheless, there is still a lack of diversity of a socioeconomic and environmental statuses of the countries where the studies are performed.

To obtain more reliable and accurate data, a large sample size is required. Nationwide databases of claim records are an adequate solution for this problem, but lack detailed information about the injury environments. Conversely, institutional data based on single or multiple hospitals or institutions, can provide more detailed information about the injury environments, but lack representativeness. Therefore, a complementary approach using both types of data is necessary.

This study investigated the incidence, characteristics, and trends of pediatric traumatic injuries to facilitate the establishment of preventive measures, such as educational programs, to improve the safety of children. For this purpose, we analyzed and compared both nationwide-sample cohort and single-institutional data.

## Materials and methods

### Datasets

Two different databases were retrospectively reviewed for this study; both were established on the basis of the International Classification of Diseases version 10. The present study protocol was reviewed and approved by the Institutional Review Board of the Asan Medical Center, Seoul, South Korea (approval no. 2017–0384).

#### 1. Nationwide-sample data

Nationwide-sample data was obtained from the Korean National Health Insurance Sharing Service (NHISS). The Korean National Health Insurance Program covers the entire Korean population (97% of the population has health insurance and 3% has medical aid), and its database has been used for many epidemiological studies [13–16]. From the database, NHISS has released random nationwide-sample cohort data that are stratified according to age, sex, region, and other variables annually since 2002 (http://nhiss.nhis.or.kr). The data consist of the claim records of approximately 1 million patients each year [13]. In the released data, age is categorized in 5-year increments to avoid revealing sensitive patient information.

#### 2. Single-institutional data

Our hospital has a dedicated pediatric emergency department (ED), which was opened in December 2010; prior to this, pediatric patients were treated in the same ED as adult patients. A biomedical research system is available at our hospital, which includes a search function that allows specified keywords to be identified in the electronic medical records [17, 18].

### Patients

#### 1. Nationwide-sample data

Cases released in the NHISS dataset between January 1, 2006 and December 31, 2015 were assessed for eligibility. Patients aged <15 years who were identified with an S-code diagnosis (denoting traumatic injury) and initially treated by an emergency medicine specialist were included. Of 10,114,909 released cases, 35,064 (0.3%) were included in the analyses.

#### 2. Single-institutional data

Patients aged <15 years who visited our ED over the same 10-year period as the nationwide-sample data and who were identified with an S-code diagnosis were included in the single-institutional dataset. A total of 39,228 patients were included in the analyses.

The number of included patients from each data source per year and corresponding national annual population statistics of South Korea are presented in Table 1.

**Table 1:** The numbers of pediatric traumatic injury cases in the NHISS and single-institutional data, and national population statistical data by year.

Year	NHISS data		Single-institutional data	National statistical data†	
	Number of released cases*	Number of included cases**	Number of included cases**	Total Korean population	Korean population aged 0–14 years (%)
2006	996,393	2,421	2,560	48,438,292	8,979,585 (18.5%)
2007	1,005,549	2,977	2,176	48,683,638	8,714,382 (17.9%)
2008	1,008,693	3,150	1,971	49,054,708	8,478,823 (17.3%)
2009	1,012,695	3,406	2,973	49,307,835	8,229,264 (16.7%)
2010	1,013,299	3,690	2,704	49,554,112	7,979,439 (16.1%)
2011	1,013,297	3,807	3,669	49,936,638	7,771,460 (15.6%)
2012	1,015,567	3,779	4,940	50,199,853	7,577,231 (15.1%)
2013	1,015,049	3,993	5,558	50,428,893	7,392,237 (14.7%)
2014	1,017,343	4,136	6,552	50,746,659	7,213,693 (14.2%)
2015	1,017,024	3,705	6,125	51,014,947	7,029,883 (13.8%)
Total	10,114,909	35,064	39,228	497,365,575	79,365,997 (16.0%)

NHISS, National Health Insurance Sharing Service.

*Approximately 1 million cases are released by NHISS each year.

**Only patients aged <15 years who received an S-code diagnosis at emergency departments were included.

†Data were obtained from the Korean Statistical Information Service (http://kosis.kr/index/index.do).

### Investigated variables

From the NHISS dataset, sex, age group (0–4, 5–9, or 10–14 years), medical provider department (e.g., emergency medicine, orthopedic surgery, or internal medicine), diagnostic code, and claim date were investigated. For patients with >1 claim record with an S-code, only the first entry was included.

From the single-institutional data, sex, age, diagnostic code, claim date, date of ED visit, place of injury (location where the injury occurred, classified as home, public place, school, restaurant, medical center, and others), and specific circumstances of the injury were investigated. For patients with >1 claim record with an S-code, only the first entry was included, and the age at the earliest claim was adopted.

Trends over time and characteristics of the injuries were investigated using both datasets; the type of injury and affected body part were determined using diagnostic codes. The timing of the ED visit (e.g., month, weekday, holiday) was determined using the claim date. In addition, detailed data about the injury environments were investigated using the single-institutional dataset; specific circumstances of the injury were evaluated using the following terminology: outdoor playground; kids cafe (indoor playground); school; private educational institute; trampoline; martial arts, such as taekwondo (Korean martial art), kendo (Japanese fencing), judo, or kung fu; sports such as skiing, snowboarding, sledding, or skating; and the use of specific equipment such as slides, swings, scooters, or wheeled sneakers. To select the appropriate term for each injury environment, and to cover the possibility of common spelling errors, the search terms were selected after reviews of a random sample of 1,000 cases.

### Data analyses

The annual incidence rates per 1 million claim cases each year were presented for the NHISS data. To determine age-specific standardization by year, data on the entire Korean population in 2006, which were available from the Korean Statistical Information Service (http://kosis.kr), were established as the standard population.

A linear regression model was used to evaluate the trends in incidences by year. Statistical analyses were conducted using SPSS for Windows (version 21; IBM Co., Armonk, NY, USA) and p-values <0.05 were considered significant.

## Results

### Comparisons between the NHISS and single-institutional data

**Fig 1:**
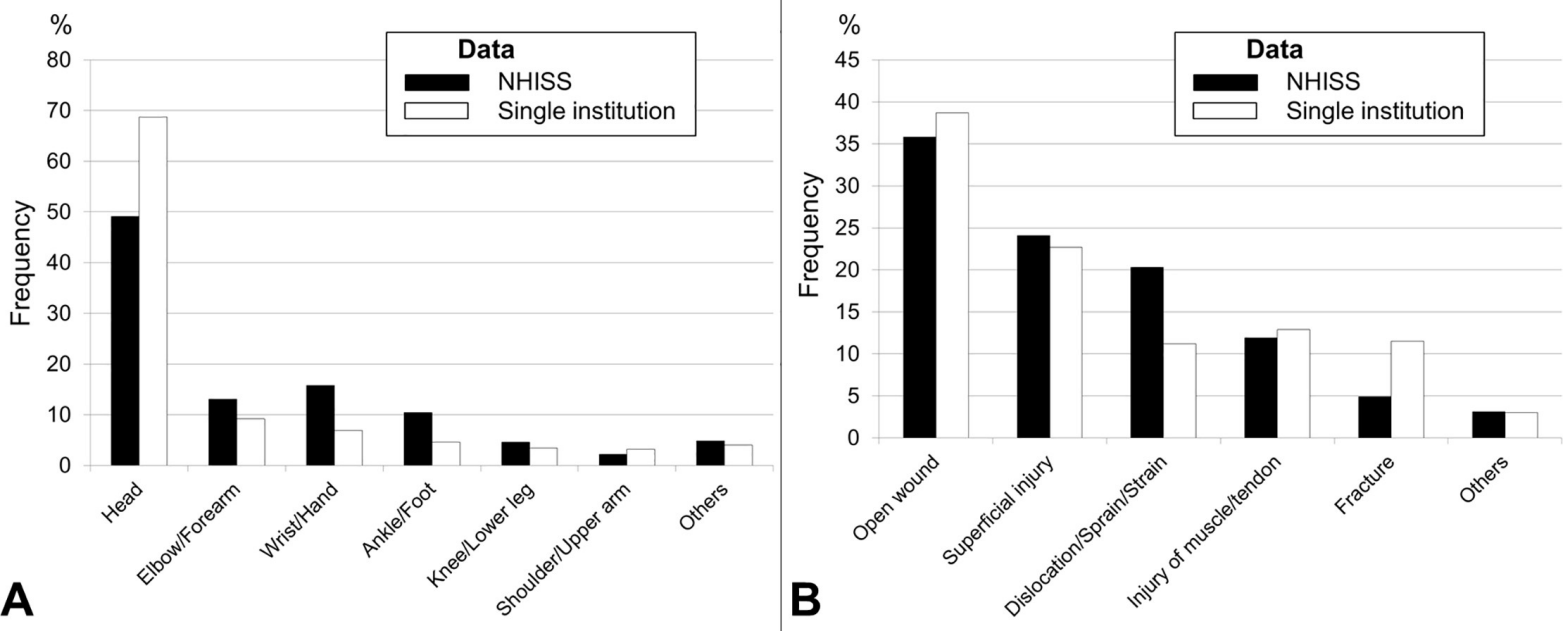
(A) Injured body part. In both the Korean National Health Insurance Sharing Service and single-institutional data, the most commonly injured body part was the head, followed by the upper extremities distal to the elbow (that is, the elbow to hand) and the lower extremities distal to the knee (that is, the knee to foot). Injuries of the trunk or proximal extremities such as the shoulders or hips were rare. (B) Injury type. Low-energy injuries such as open wounds or superficial injuries were most common in both datasets.

Table 2 shows demographic comparisons between the NHISS and single-institutional data. There were no significant differences in sex, age distribution, injured body part (Fig 1A), injury type (Fig 1B), and visit pattern between the datasets.

**Table 2:** Comparisons between the single-institutional and NHISS data.

	Single-institutional data	NHISS data
Sex, n (%)		
Male	24,640 (62.8%)	21,644 (61.7%)
Female	14,588 (37.2%)	13,420 (38.3%)
Age, n (%)		
0–4 years	24,700 (63.0%)	17,479 (49.8%)
5–9 years	9,204 (23.5%)	10,272 (29.3%)
10–14 years	5,316 (13.6%)	7,313 (20.9%)
Injured body part, n (%)		
Head	26,931 (68.7%)	17,230 (49.1%)
Wrist/hand	2,708 (6.9%)	5,544 (15.8%)
Elbow/forearm	3,616 (9.2%)	4,595 (13.1%)
Ankle/foot	1,798 (4.6%)	3,640 (10.4%)
Knee/lower leg	1,337 (3.4%)	1,619 (4.6%)
Shoulder/upper arm	1,260 (3.2%)	756 (2.2%)
Others	1,578 (4.0%)	1,680 (4.8%)
Injury type, n (%)		
Open wound	15,191 (38.7%)	12,548 (35.8%)
Superficial injury	8,890 (22.7%)	8,455 (24.1%)
Dislocation/sprain/strain	4,399 (11.2%)	7,107 (20.3%)
Injury of muscle/tendon	5,049 (12.9%)	4,171 (11.9%)
Fracture	4,521 (11.5%)	1,702 (4.9%)
Others	1,178 (3.0%)	1,081 (3.1%)
Number of visits by month, n (%)		
January–February	4,933 (12.6%)	4,765 (13.6%)
March–April	6,540 (16.7%)	5,953 (17.0%)
May–June	7,465 (19.0%)	7,211 (20.6%)
July–August	7,172 (18.3%)	6,246 (17.8%)
September–October	7,285 (18.6%)	5,859 (16.7%)
November–December	5,833 (14.9%)	5,030 (14.3%)
Injuries by day of the week, n (n/day)		
Weekday	24,517 (9.4/day)	20,123 (7.7/day)
Weekend	14,711 (14.1/day)	14,941 (14.3/day)
Ratio of weekend/weekday injuries per day	1.50	1.86
Injuries by day of the year, n (n/day)		
Working day	22,992 (9.2/day)	18,407 (7.4/day)
Holiday	16,236 (14.1/day)	16,657 (14.5/day)
Ratio of holiday/working day injuries per day	1.53	1.96

NHISS, National Health Insurance Sharing Service. There were no significant differences in the characteristics between the datasets.

### Changes over time in the NHISS and single-institutional data

**Fig 2:**
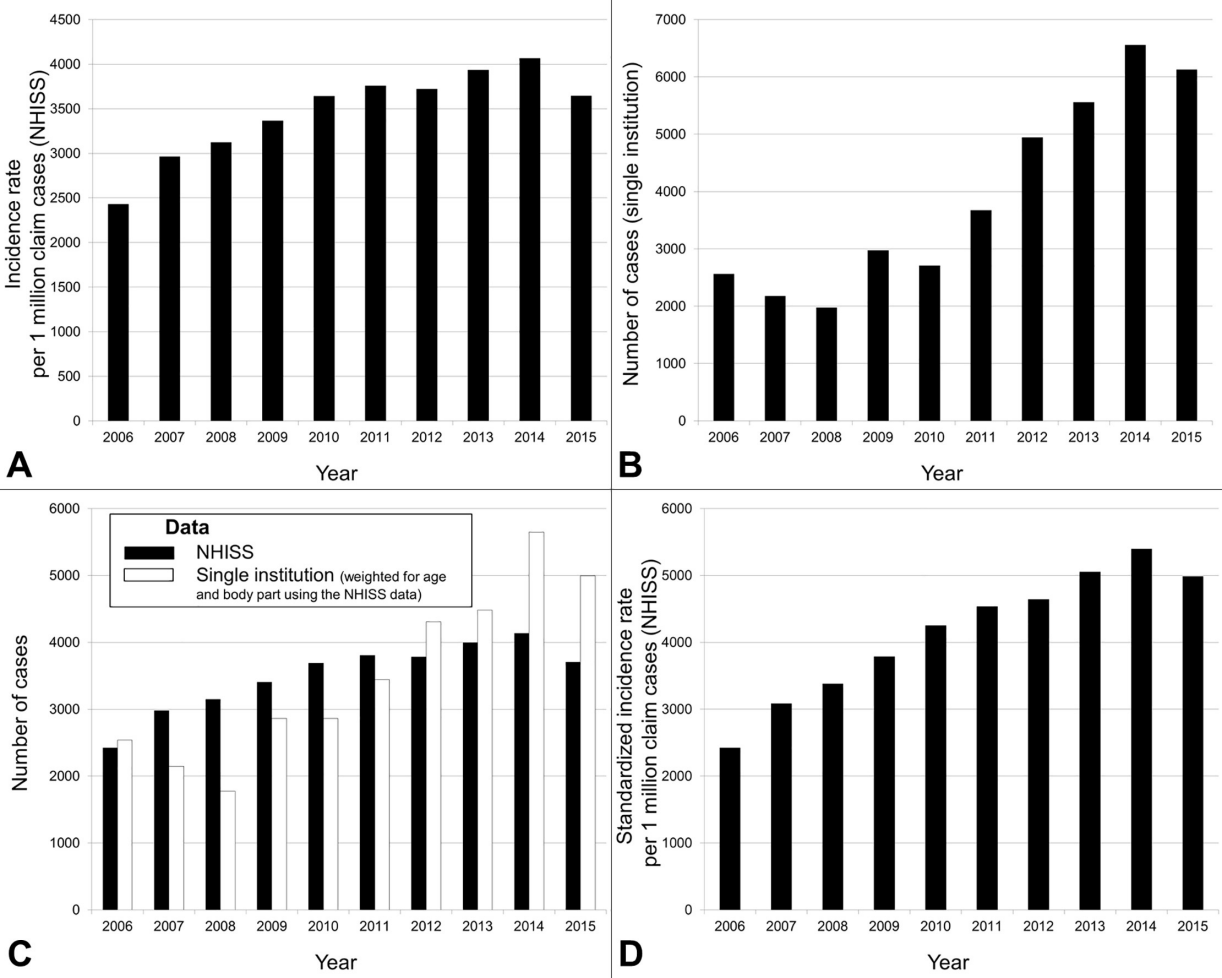
(A) Korean National Health Insurance Sharing Service (NHISS) data for the injury incidence rate per 1 million claim cases according to year. The incidence rate tended to increase during the 10-year study period (β = 0.877, p = 0.001). The decrease in 2015 may be explained by the outbreak of Middle East respiratory syndrome, when there was an attempt to reduce emergency department visits to prevent the spread of the virus. (B) Single-institutional data for visiting patients by year. The number of injured patients tended to increase over the study period (β = 0.933, p < 0.001) with more rapid changes occurring after 2010, which coincides with the opening of a pediatric emergency center in December of that year. (C) When the numbers of cases of single-institutional data were weighted for age and body part using the NHISS data, there was a greater increase in the single-institutional data. (D) NHISS data for the standardized injury incidence rate per 1 million claim cases by year. Although there appeared to be a greater increase in the single-institutional data in Fig 2C, the rate of increase in the NHISS data became more rapid after standardization to the population composition in 2006 (β = 0.964, p < 0.001). Despite a decreased proportion of children aged 0–14 years, the injury incidence rate increased over time. Therefore, the rate of pediatric traumatic injuries may have increased more rapidly than assumed.

Although the proportion of children aged 0–14 years in the overall Korean population gradually declined during the study period, the frequency of traumatic injuries tended to increase over time in both datasets (β = 0.877, p = 0.001 in the NHISS dataset and β = 0.933, p < 0.001 in the single-institutional data; Fig 2A, 2B and 2C). The incidences decreased abruptly in 2015 in both datasets. When the incidence rates of the NHISS data were standardized to the national population in 2006 (Table 1), the rate of increase was accelerated (β = 0.964, p < 0.001; Fig 2D).

### Detailed data about the injury environments (by single-institutional data

**Fig 3:**
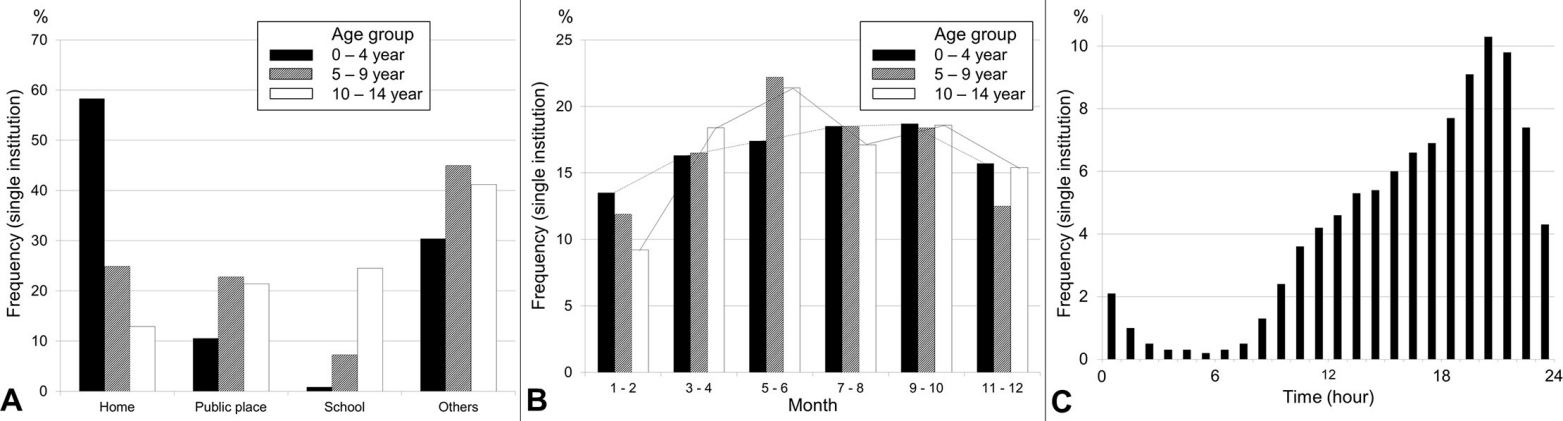
(A) Place of injury by age. The home was the most common location where injury occurred in young children. As age increased, the injury rates outside the home also increased. (B) Monthly visit frequencies by age. The differences in the frequencies of emergency department visits by month became more apparent as age increased. For older children, the visit frequency peaked in May and June, which is a period suitable for outdoor activities. By contrast, the frequency of visits was lowest during winter. (C) Time of emergency department visits. The most common time when emergency departments were visited was between 7 and 10 pm.

**Fig 4:**
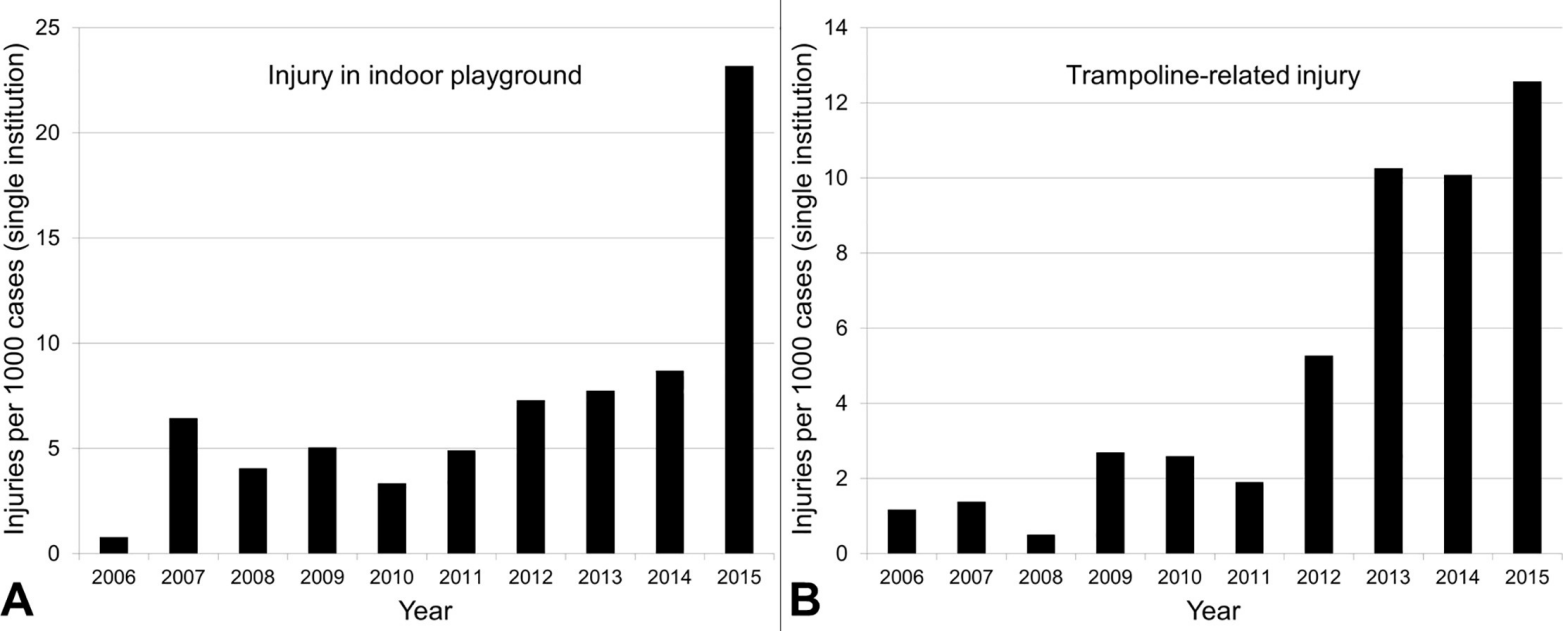
The proportions of indoor playground (A) and trampoline-related (B) injuries increased rapidly during the study period.

We examined the injury data in the single-institutional cohort on the basis of the affected body part (Table 3), injury type (Table 4), diagnostic code (Table 5), place of injury (Fig 3A), and ED visit patterns by month (Fig 3B). The most common time of ED visits was 7–10 pm (Fig 3C). Among the variables that were investigated regarding the injury environments, the frequencies of indoor playground and trampoline-related injuries increased rapidly during the study period (Fig 4).

**Table 3:** Single-institutional data regarding injured body parts by age.

Age group (years)	Order of frequency			
	1st	2nd	3rd	4th
0	Head	Elbow/forearm †	Wrist/hand †	Shoulder/upper arm
	2759 (82.2%)	281 (8.4%)	151 (4.5%)	77 (2.3%)
1–2	Head	Elbow/forearm †	Wrist/hand †	Ankle/foot ‡
	9944 (73.4%)	1524 (11.2%)	968 (7.1%)	429 (3.2%)
3–4	Head	Elbow/forearm †	Wrist/hand †	Ankle/foot ‡
	5480 (70.3%)	805 (10.3%)	466 (6.0%)	306 (3.9%)
5–6	Head	Elbow/forearm †	Ankle/foot ‡	Wrist/hand †
	3107 (66.7%)	330 (7.1%)	305 (6.6%)	263 (5.6%)
7–8	Head	Ankle/foot ‡	Wrist/hand †	Elbow/forearm †
	2098 (63.7%)	223 (6.8%)	220 (6.7%)	195 (5.9%)
9–10	Head	Knee/lower leg ‡	Wrist/hand †	Ankle/foot ‡
	1317 (56.6%)	199 (8.6%)	191 (8.2%)	179 (7.7%)
11–12	Head	Wrist/hand †	Knee/lower leg ‡	Ankle/foot ‡
	1103 (51.0%)	248 (11.5%)	183 (8.5%)	180 (8.3%)
13–14	Head	Knee/lower leg ‡	Wrist/hand †	Elbow/forearm †
	1123 (54.0%)	208 (10.0%)	201 (9.7%)	145 (7.0%)
Total	Head	Elbow/forearm †	Wrist/hand †	Ankle/foot ‡
	26931 (68.7%)	3616 (9.2%)	2708 (6.9%)	1798 (4.6%)

The head was the most frequently injured body part, whereas the trunk and proximal extremities were rarely injured across all age groups. Upper extremity injuries distal to the elbow (†) were more frequent than lower-extremity injuries distal to the knee (‡) in younger children, but became less frequent in older children. Data are presented as n (%).

**Table 4:** Single-institutional data regarding injury type by age.

Age group (years)	Order of frequency			
	1st	2nd	3rd	4th
0	Superficial injury	Muscle/tendon injury	Open wound	Dislocation/sprain/strain
	1065 (31.7%)	1016 (30.3%)	555 (16.5%)	328 (9.8%)
1–2	Open wound	Superficial injury	Dislocation/sprain/strain	Muscle/tendon injury
	6104 (45.0%)	3080 (22.7%)	1841 (13.6%)	1545 (11.4%)
3–4	Open wound	Superficial injury	Dislocation/sprain/strain	Muscle/tendon injury
	3522 (45.2%)	1641 (21.0%)	937 (12.0%)	816 (10.5%)
5–6	Open wound	Superficial injury	**Fracture**	Muscle/tendon injury
	1971 (42.3%)	1025 (22.0%)	633 (13.6%)	473 (10.2%)
7–8	Open wound	Superficial injury	**Fracture**	Muscle/tendon injury
	1286 (39.0%)	701 (21.3%)	551 (16.7%)	371 (11.3%)
9–10	Open wound	Superficial injury	**Fracture**	Muscle/tendon injury
	729 (31.3%)	520 (22.4%)	497 (21.4%)	303 (13.0%)
11–12	**Fracture**	Open wound	Superficial injury	Muscle/tendon injury
	588 (27.2%)	556 (25.7%)	452 (20.9%)	265 (12.3%)
13–14	**Fracture**	Open wound	Superficial injury	Muscle/tendon injury
	620 (29.8%)	468 (22.5%)	406 (19.5%)	260 (12.5%)
Total	Open wound	Superficial injury	Muscle/tendon injury	Fracture
	15191 (38.7%)	8890 (22.7%)	5049 (12.9%)	4521 (11.5%)

Low-energy injuries (that is, superficial injuries such as abrasions or open wounds such as lacerations) were the most common injury type for children aged <5 years. Although fracture is a rare injury type in young children, it was relatively common in children aged >5 years and became the most common injury type in children aged >11 years (bold font). Data are presented as n (%).

**Table 5:** Single-institutional data regarding diagnostic codes by age.

Age group (years)	Order of frequency					
	1st	2nd	3rd	4th	5th	6th
0	S06	S00	S01	S53	S02	S60
	1016 (30.3%)	937 (27.9%)	490 (14.6%)	267 (8.0%)	230 (6.9%)	62 (1.8%)
1–2	S01	S00	S06	S53	S60	S61
	5744 (42.4%)	2093 (15.4%)	1542 (11.4%)	1358 (10.0%)	515 (3.8%)	242 (1.8%)
3–4	S01	S00	S06	S53	S60	S42
	3274 (42.0%)	987 (12.7%)	811 (10.4%)	583 (7.5%)	242 (3.1%)	199 (2.6%)
5–6	S01	S00	S06	S52	S42	S02
	1745 (37.5%)	570 (12.2%)	461 (9.9%)	182 (3.9%)	178 (3.8%)	141 (3.0%)
7–8	S01	S00	S06	S02	S52	S42
	1096 (33.3%)	361 (11.0%)	358 (10.9%)	144 (4.4%)	143 (4.3%)	107 (3.2%)
9–10	S01	S06	S00	S02	S52	S42
	583 (25.1%)	285 (12.3%)	232 (10.0%)	125 (5.4%)	124 (5.3%)	91 (3.9%)
11–12	S01	S06	S00	S02	S52	S62
	409 (18.9%)	254 (11.8%)	187 (8.7%)	161 (7.5%)	136 (6.3%)	90 (4.2%)
13–14	S01	S02	S06	S00	S52	S82
	331 (15.9%)	250 (12.0%)	244 (11.7%)	185 (8.9%)	111 (5.3%)	79 (3.8%)
Total	S01	S00	S06	S53	S02	S60
	13672 (34.9%)	5552 (14.2%)	4971 (12.7%)	2357 (6.0%)	1423 (3.6%)	1138 (2.9%)

Open wounds (S01), superficial injuries (S00), and muscle/tendon injuries (S06) of the head were the most frequent injuries in nearly all age groups, but decreased with increasing age (72.8% at <1 year, 69.2% at 1–2 years, 65.1% at 3–4 years, and 36.5% at 13–14 years). Notably, superficial injuries and open wounds of the head comprised approximately one-half of all injuries in children aged <8 years (42.5% at <1 year, 57.8% at 1–2 years, 54.7% at 3–4 years, 49.7% at 5–6 years, and 44.3% at 7–8 years). S53, dislocation/sprain/strain of elbow/forearm; S02, fracture of head; S60, superficial injury of wrist/hand; S61, open wound of wrist/hand; S42, fracture of shoulder/upper arm; S52, fracture of elbow/forearm; S62, fracture of wrist/hand; S82, fracture of knee/lower leg. Data are presented as n (%).

## Discussion

We analyzed and compared nationwide-sample (NHISS) and single-institutional data to investigate the characteristics and trends of pediatric traumatic injuries. The datasets each had advantages and disadvantages as follows: the NHISS data reflected national trends, but did not provide detailed information; by contrast, single-institutional data presented detailed information about the injury environments, but these were not representative nationally. We believe that our complementary approach using both datasets overcomes these disadvantages. The present results will help to establish preventive measures, such as educational programs, to improve the safety of children.

### Similarities between the datasets

The NHISS and single-institutional data were similar in many regards (Table 2). Both datasets revealed that injuries were more frequent in boys and younger children (aged 0–4 years). The most frequently injured body part was the head, followed by the upper extremities distal to the elbow (elbow to hand) and the lower extremities distal to the knee (knee to foot). The most common types of injury were open wounds and lacerations (Fig 1). Pediatric trauma patients visited the ED 1.5–2-fold more frequently on holidays and weekends than on working days and weekdays (Table 2). These results suggest that the injury/medical environments associated with pediatric trauma are relatively similar throughout the country; therefore, the detailed results of single-institutional datasets could be generalized to national circumstances with adequate external validity.

### Increasing frequency of injuries in children despite a declining pediatric population

South Korea has experienced rapid decreases in both mortality and fertility, which have contributed to increasing the population age [19]; consequently, the proportion of children (aged 0–14 years) among the whole population is decreasing (Table 1). However, the incidences of pediatric traumatic injury in both datasets increased over time (Fig 2). Therefore, the standardized NHISS data revealed more rapid increases than the unstandardized data. More effective preventive measures that are strictly adhered to are necessary to address the rapid growth in the incidence of pediatric traumatic injuries over the past 10 years.

The more rapid increases in the incidences of injury after 2011 in the single-institutional data might be partially attributable to the opening of a pediatric emergency center in December 2010. The decreases in 2015 in both datasets may be attributable to the national recommendation to avoid ED visits where possible to suppress the outbreak of Middle East respiratory syndrome [20].

To assess the cause of the rapid increase observed during the 10-year study period, we surveyed the environment at the time of injury. Among the surveyed variables, the proportions of indoor playgrounds and trampoline-related injuries increased rapidly (Fig 4). The increased number of trampolines in many indoor playgrounds or trampoline parks could be a contributing factor [21]. Although trampolines are widely available in the US, the frequency of trampoline-related injuries has somewhat declined, as guidelines for their use have become broadly acknowledged [22]. However, the frequency of trampoline-related injuries has continued to increase in many other countries [23–25]. Thus, the national promotion of guidelines to prevent trampoline-related injuries, especially in public areas, is necessary.

### Commonly injured body parts

The head was the most frequently injured body part, followed by the upper extremities distal to the elbow and the lower extremities distal to the knee (Fig 1A). Although the frequency changed with age, the trunk and proximal extremities, such as the shoulder and hip, were rarely injured across all age groups (Tables 3 and 5). Therefore, to prevent traumatic injuries in children, it may be more effective to wear protective equipment that covers the head and the extremities distal to the elbow and knee, rather than the trunk or proximal extremities.

### Characteristics of injuries in preschoolers

In children aged <5 years, the head was the most frequently injured body part followed by the upper extremities distal to the elbow (Table 3), and low-energy injuries (that is, superficial injuries such as abrasions or open wounds such as lacerations) were the most common injury type (Table 4). Indeed, superficial injuries and open wounds of the head comprised approximately one-half of all injuries in children aged <8 years (Table 5). Therefore, many injuries could be prevented in this age group by wearing simple clothing that covers the head, such as a cap or hat.

The diagnostic code S53 (which denotes a dislocation, sprain, or strain of the elbow or forearm) was common in children aged <5 years, and may be explained in part by the high incidence of pulled elbows in this age group [26, 27].

### Characteristics of injuries in children aged ≥ 5 years

The rate of lower-extremity injuries was higher in older children (Table 3). Foot and ankle injuries were more common in children aged <9 years, whereas knee and lower leg injuries were more common in those aged ≥ 9 years. However, these findings do not suggest that older children have a lower risk for foot and ankle injuries. The frequency of foot and ankle injuries was maintained consistently across the age groups, comprising 6%–8% of injuries in those aged 7 to approximately 12 years. Fracture was relatively common after the age of 5 years, and was the most common injury type in children aged >11 years (Table 4). Considering that fractures affecting the lower extremities are often associated with sports or leisure activities [28, 29], findings suggest that lower-extremity injuries become more common as children grow older because of increased participation in such activities. This assumption is supported by the finding that older children exhibited an increased rate of injuries outside the home (Fig 3A) and a large increase in the frequency of injuries during May and June, which are suitable months for outdoor activities in authors’ country because there are many holidays and the temperature is adequate (Fig 3B).

Although a diagnostic code of S53 was relatively common in children aged <5 years of age, S52 (which denotes a fracture of elbow or forearm) was relatively common in older children (Table 5), probably as a result of increases in the supracondylar and lateral condylar humeral fractures in this age group [30–32].

### Timing of ED visits

Visits to the ED peaked between 7 and 10 pm (Fig 3C). This finding could indicate that pediatric injuries frequently occur in this period; however, it could also be a result of patients visiting the outpatient clinic rather than the ED during earlier periods (9 am to 5 pm) or be due to difficulties among parents in accessing healthcare facilities given the high proportion of young working couples in authors’ country (http://www.index.go.kr/potal/main/EachDtlPageDetail.do?idx_cd=3037). These same interpretations could be used to explain the higher number of visits on holidays and weekends in both datasets (Table 2). More research is needed to determine the cause underlying the increased frequency of visits during the evening or on holidays; however, it is clear that sufficient numbers of emergency medical personnel should be made available to attend to pediatric trauma cases during these busy periods.

### Limitations

Some considerations and limitations should be noted when interpreting the results of the present study. First, the medical environments for trauma differ by country; therefore, it is difficult to determine whether these data are generalizable outside of South Korea. Nevertheless, as the general lifestyle and medical environment become more similar globally [1], results of the present study are likely to become applicable to many countries with similar socioeconomic conditions to ours. Second, although the NHISS and single-institutional data were similar in many aspects, it is necessary to recognize the possibility of selection bias caused by referrals of more severe cases to our institute, which is a tertiary referral hospital. Third, there may be limitations regarding the use of sample data. The sample cohort used in the present study were randomly selected and stratified data reflecting approximately 1 million claims records per year; therefore, the sample data may not reflect medical utilization among the total population, and further comprehensive studies may be needed.

## Conclusions

To investigate the characteristics and trends of pediatric traumatic injuries, we used a complementary approach including both nationwide-sample and single-institutional data. Based on the similarities between both datasets, the detailed results of the single-institutional data could be extended and applied nationally with adequate external validity. Despite the declining pediatric population, the incidence of pediatric traumatic injuries has increased, including the proportions of indoor playground and trampoline-related injuries. The increase of pediatric traumatic injuries may be partially a result of the increased availability of indoor playgrounds and installation of trampolines. Preventive guidelines have been published, but stricter adherences to their use would be needed. The most commonly injured body part was the head, followed by the upper extremities distal to the elbow (elbow to hand) and the lower extremities distal to the knee (knee to foot). Injuries of the trunk or proximal extremities such as the shoulders or hips were rare. To prevent traumatic injuries in children, it may be more effective to wear protective gear covering the head and extremities distal to the elbow or knee, rather than the trunk or proximal extremities. For preschoolers (aged <5 years), low-energy injuries of the head were most common; therefore, simple clothing such as caps could prevent many injuries in this age group. Among children aged ≥ 5 years, the rates of lower-extremity injuries, fractures, and injuries occurring outside the home increased, and seasonal variation (peak incidence in May and June) became evident. Protective guidelines for outdoor sports and leisure activities are necessary for these children. Visits to the ED peaked between 7 and 10 pm, and on holidays and weekends. Policies to increase medical support for pediatric trauma in the ED during these periods should be considered. The present results could facilitate the establishment of preventive measures, such as educational programs, to improve the safety of children.

## Supporting information

S1 File Data of the single-institutional data. (SAV)

S2 File Data of the nationwide sample data. (SAV)

## References

[1] Alonge O, Khan UR, Hyder AA. Our Shrinking Globe: Implications for Child Unintentional Injuries. Pediatr Clin North Am. 2016;63(1):167–81. 10.1016/j.pcl.2015.08.009 26613695

[2] Johnston BD, Ebel BE. Child injury control: trends, themes, and controversies. Acad Pediatr. 2013;13(6):499–507. 10.1016/j.acap.2013.04.016 24021529

[3] Booth VM, Rowlands AV, Dollman J. Physical activity temporal trends among children and adolescents. J Sci Med Sport. 2015;18(4):418–25. 10.1016/j.jsams.2014.06.002 25041963

[4] Eithsdottir ST, Kristjansson AL, Sigfusdottir ID, Allegrante JP. Trends in physical activity and participation in sports clubs among Icelandic adolescents. Eur J Public Health. 2008;18(3):289–93. 10.1093/eurpub/ckn004 18285360

[5] Salmon J, Timperio A, Cleland V, Venn A. Trends in children's physical activity and weight status in high and low socio-economic status areas of Melbourne, Victoria, 1985–2001. Aust N Z J Public Health. 2005;29(4):337–42. 16222931 10.1111/j.1467-842x.2005.tb00204.x

[6] Centers for Disease C, Prevention. Participation in high school physical education—Ontario, Canada, 1999–2005. MMWR Morb Mortal Wkly Rep. 2007;56(3):52–4. 17251898

[7] Strong WB, Malina RM, Blimkie CJ, Daniels SR, Dishman RK, Gutin B, et al Evidence based physical activity for school-age youth. J Pediatr. 2005;146(6):732–7. 10.1016/j.jpeds.2005.01.055 15973308

[8] Felfe C, Lechner M, Steinmayr A. Sports and Child Development. PLoS One. 2016;11(5):e0151729 10.1371/journal.pone.0151729 27144474 PMC4856309

[9] Landry BW, Driscoll SW. Physical activity in children and adolescents. PM R. 2012;4(11):826–32. 10.1016/j.pmrj.2012.09.585 23174545

[10] McDonald NC, Brown AL, Marchetti LM, Pedroso MS. U.S. school travel, 2009 an assessment of trends. Am J Prev Med. 2011;41(2):146–51. 10.1016/j.amepre.2011.04.006 21767721

[11] Pearson M, Hunt H, Garside R, Moxham T, Peters J, Anderson R. Preventing unintentional injuries to children under 15 years in the outdoors: a systematic review of the effectiveness of educational programs. Inj Prev. 2012;18(2):113–23. 10.1136/injuryprev-2011-040043 21890579 PMC3311869

[12] Hashikawa AN, Newton MF, Cunningham RM, Stevens MW. Unintentional injuries in child care centers in the United States: a systematic review. J Child Health Care. 2015;19(1):93–105. 10.1177/1367493513501020 24092867

[13] Shin J, Choi Y, Lee SG, Kim TH, Park EC. Higher cost sharing for visiting general hospitals and the changing trend in the first-visited healthcare organization among newly diagnosed hypertension patients. Medicine (Baltimore). 2016;95(40):e4880. 27749543 10.1097/MD.0000000000004880 PMC5059045

[14] Park SJ, Choi NK, Park KH, Woo SJ. Five year nationwide incidence of rhegmatogenous retinal detachment requiring surgery in Korea. PLoS One. 2013;8(11):e80174 10.1371/journal.pone.0080174 24236173 PMC3827446

[15] Kang HY, Yang KH, Kim YN, Moon SH, Choi WJ, Kang DR, et al Incidence and mortality of hip fracture among the elderly population in South Korea: a population-based study using the national health insurance claims data. BMC Public Health. 2010;10:230 10.1186/1471-2458-10-230 20438644 PMC2874780

[16] Gong HS, Oh WS, Chung MS, Oh JH, Lee YH, Baek GH. Patients with wrist fractures are less likely to be evaluated and managed for osteoporosis. J Bone Joint Surg Am. 2009;91(10):2376–80. 10.2106/JBJS.H.01871 19797572

[17] Shin SY, Lyu Y, Shin Y, Choi HJ, Park J, Kim WS, et al Lessons Learned from Development of De-identification System for Biomedical Research in a Korean Tertiary Hospital. Healthc Inform Res. 2013;19(2):102–9. 10.4258/hir.2013.19.2.102 23882415 PMC3717433

[18] Shin Y, Choi C, Lee J, Shin SY. First Step to Big Data Research in Hospital. Stud Health Technol Inform. 2015;216:924 26262226

[19] Dronina Y, Yoon YM, Sakamaki H, Nam EW. Health System Development and Performance in Korea and Japan: A Comparative Study of 2000–2013. J Lifestyle Med. 2016;6(1):16–26. 10.15280/jlm.2016.6.1.16 27358836 PMC4915763

[20] Jack A. Why the panic? South Korea's MERS response questioned. BMJ. 2015;350:h3403 10.1136/bmj.h3403 26108610

[21] Choi ES, Hong JH, Sim JA. Distinct features of trampoline-related orthopedic injuries in children aged under 6 years. Injury. 2018;49(2):443–6. 10.1016/j.injury.2017.12.017 29273293

[22] Council on Sports M, Fitness AAoP, Briskin S, LaBotz M. Trampoline safety in childhood and adolescence. Pediatrics. 2012;130(4):774–9. 10.1542/peds.2012-2082 23008455

[23] Cheung MY, Lai CL, Lam WH, Lau JS, Lee AK, Yuen GG, et al Trampoline-related injuries in Hong Kong. Hong Kong Med J. 2016;22(1):81–4. 10.12809/hkmj144411 26845468

[24] Ashby K, Pointer S, Eager D, Day L. Australian trampoline injury patterns and trends. Aust N Z J Public Health. 2015;39(5):491–4. 10.1111/1753-6405.12404 26123781

[25] Konigshausen M, Gothner M, Kruppa C, Dudda M, Godry H, Schildhauer TA, et al [Trampoline-related injuries in children: an increasing problem]. Sportverletz Sportschaden. 2014;28(2):69–74. 10.1055/s-0034-1366544 24963737

[26] Jongschaap HC, Youngson GG, Beattie TF. The epidemiology of radial head subluxation ('pulled elbow') in the Aberdeen city area. Health Bull (Edinb). 1990;48(2):58–61. 2332335

[27] Welch R, Chounthirath T, Smith GA. Radial Head Subluxation Among Young Children in the United States Associated With Consumer Products and Recreational Activities. Clin Pediatr (Phila). 2017;56(8):707–15. 28589762 10.1177/0009922816672451

[28] Su AW, Larson AN. Pediatric Ankle Fractures: Concepts and Treatment Principles. Foot Ankle Clin. 2015;20(4):705–19. 10.1016/j.fcl.2015.07.004 26589088 PMC4912125

[29] Palmu SA, Auro S, Lohman M, Paukku RT, Peltonen JI, Nietosvaara Y. Tibial fractures in children. A retrospective 27-year follow-up study. Acta Orthop. 2014;85(5):513–7. 10.3109/17453674.2014.916489 24786903 PMC4164870

[30] Khoshbin A, Leroux T, Wasserstein D, Wolfstadt J, Law PW, Mahomed N, et al The epidemiology of paediatric supracondylar fracture fixation: a population-based study. Injury. 2014;45(4):701–8. 10.1016/j.injury.2013.10.004 24183392

[31] Houshian S, Mehdi B, Larsen MS. The epidemiology of elbow fracture in children: analysis of 355 fractures, with special reference to supracondylar humerus fractures. J Orthop Sci. 2001;6(4):312–5. 10.1007/s0077610060312 11479758

[32] Kang S, Park SS. Predisposing Effect of Elbow Alignment on the Elbow Fracture Type in Children. J Orthop Trauma. 2015;29(8):e253–8. 10.1097/BOT.0000000000000322 25756916

